# Acute Acalculous Cholecystitis in Patients With Clear Cell Renal Cell Carcinoma Treated With Sunitinib: Report of Two Cases

**DOI:** 10.14740/jocmr1850w

**Published:** 2014-05-22

**Authors:** Nobuki Furubayashi, Takahito Negishi, Yu Hirata, Kenichi Taguchi, Motonobu Nakamura

**Affiliations:** a Department of Urology, National Kyushu Cancer Center, Fukuoka, Japan; b Department of Pathology, National Kyushu Cancer Center, Fukuoka, Japan

**Keywords:** Sunitinib, Clear cell renal cell carcinoma, Acute acalculous cholecystitis, Cholecystectomy, Adverse event

## Abstract

Although sunitinib is associated with a variety of adverse events, cases of sunitinib-related acute cholecystitis have rarely been reported. We herein report two cases of sunitinib-related acute acalculous cholecystitis in patients with clear cell renal cell carcinoma. In both cases, the gallbladder was surgically removed because it was difficult to improve the patient’s condition with the cessation of sunitinib and non-surgical treatment only. Attention must be paid to the possibility of sunitinib-related acute cholecystitis, which, although uncommon, can be life-threatening.

## Introduction

Sunitinib malate (SUTENT^®^, Pfizer Inc., New York, NY, USA) is an oral, multitargeted tyrosine kinase inhibitor of vascular endothelial growth factor receptors 1-3 and platelet-derived growth factor receptors α and β [1], is widely used in the treatment of metastatic renal cell carcinoma (RCC) and gastrointestinal stromal tumors (GISTs) and is often administered in the perioperative period [2]. Although sunitinib exhibits a variety of adverse events, cases of sunitinib-related acute cholecystitis have rarely been reported. We herein report two cases of sunitinib-related acute acalculous cholecystitis in patients with clear cell RCC in whom the gallbladder was surgically removed because it was difficult to improve their condition with the cessation of sunitinib only.

## Case Report

### Case 1

**Figure 1:**
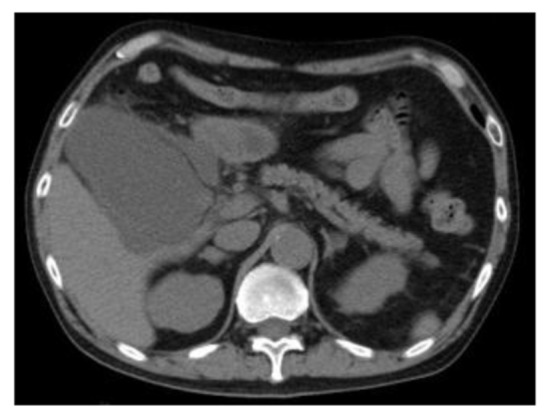
Abdominal computed tomography showed a tense and dilated gallbladder with surrounding fluid collection without gallbladder stones or emphysematous changes.

A 66-year-old Japanese male with a left renal mass detected on an examination for the cause of right buttock pain was referred to our hospital. Computed tomography (CT) showed a hypervascular and inhomogeneous tumor in the left kidney demonstrating iliopsoas muscle invasion with distant metastasis to the lungs and bone, thus suggesting left RCC of clinical T4N0M1. After confirming the histological type of the tumor to be clear cell RCC based on a percutaneous kidney biopsy, sunitinib therapy (50 mg/day, 4 weeks on and 2 weeks off) was started. During the 2 weeks of the first cycle, the patient experienced general fatigue, although no fever, right upper quadrant pain of Murphy’s sign were observed. Laboratory tests revealed elevated levels of C-reactive protein, lactate dehydrogenase, liver transaminases, alkaline phosphatase and amylase, while the white blood cell count and total bilirubin level were normal. After discontinuing the sunitinib therapy, the patient’s condition and laboratory tests improved. Two weeks later, he was readmitted for treatment with axitinib therapy and CT to evaluate the presence of pretherapeutic lesions. Despite exhibiting a normal gallbladder before sunitinib treatment, abdominal CT showed a tense and dilated gallbladder with surrounding fluid collection, but no gallbladder stones or emphysematous changes (Fig. 1), and the patient was diagnosed with acute acalculous cholecystitis. Following percutaneous transhepatic gallbladder drainage, a follow-up CT revealed that the pericholecystic fluid collection was still observed and there was no confirmation that the contrast agent from the drainage tube had passed into the common duct, and cholecystectomy was ultimately performed to control the acalculous cholecystitis.

### Case 2

**Figure 2:**
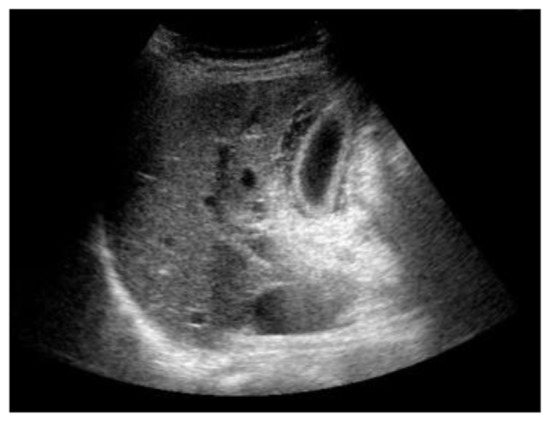
Abdominal ultrasonography revealed thickening of the gallbladder wall without gallbladder stones or emphysematous changes.

A 67-year-old Japanese female with a right renal mass detected on an examination for the cause of gross hematuria was referred to our hospital. CT and magnetic resonance imaging (MRI) showed a hypervascular and inhomogeneous tumor in the right kidney with the duodenum over Gerota’s fascia and right renal vein invasion with distant metastasis to the lungs, thus suggesting a diagnosis of right RCC of clinical T4N0M1. After confirming the histological type of the tumor to be clear cell RCC based on a percutaneous kidney biopsy, sunitinib therapy (37.5 mg/day, 4 weeks on and 2 weeks off) was started. The patient developed a fever during the 2 weeks of the first cycle; however, she exhibited no right upper quadrant pain, and Murphy’s sign was negative. Laboratory tests revealed only an elevated percentage of neutrophils, while the white blood cell count was within the normal limits. She was diagnosed with a bacterial infection, which was treated with broad-spectrum antibiotics, and the sunitinib therapy was discontinued. Three days later, laboratory tests disclosed elevated levels of C-reactive protein, total bilirubin, lactate dehydrogenase, liver transaminases, alkaline phosphatase and amylase, although the white blood cell count remained normal. Despite having a normal gallbladder prior to treatment, abdominal ultrasonography revealed thickening of the gallbladder wall without gallbladder stones or emphysematous changes (Fig. 2); therefore, the patient was diagnosed with acute acalculous cholecystitis. She recovered following treatment with fasting and antibiotics, without percutaneous transhepatic gallbladder drainage. Two weeks later, she again developed acute cholecystitis, and cholecystectomy was ultimately performed to control the acute cholecystitis.

## Discussion

We experienced two patients who underwent cholecystectomy to control acalculous cholecystitis caused by the administration of sunitinib. Although sunitinib is associated with a variety of adverse events, cases of sunitinib-related acute cholecystitis have rarely been reported. In the present two cases, it was necessary for the patients to undergo cholecystectomy in order to control acute cholecystitis.

**Figure 3:**
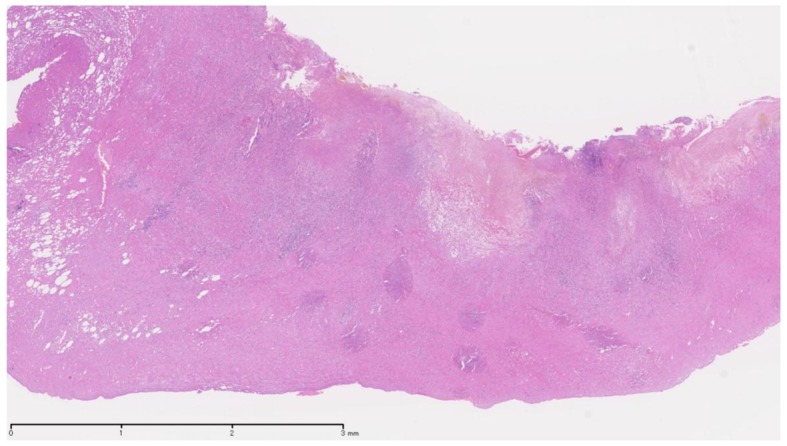
Thickened gallbladder wall in transmural lymphoeosinophilic inflammation with hemorrhage and necrotic change.

In the previous literature, only three cases of sunitinib-related acute cholecystitis have been reported, including one patient with a GIST [3] and two patients with RCC [4, 5]. The Naranjo adverse drug reaction probability scale scores for these events were 6 and 5, respectively, indicating the probable association of these events with sunitinib treatment [6]. The characteristic findings of general acute cholecystitis were not recognized in the excised gallbladders on pathology (Fig. 3). Although the exact pathologic mechanism remains uncertain, we speculate that sunitinib plays a role in the development of acalculous cholecystitis by inducing local endothelial injury and gallbladder ischemia, as judged from the mechanism of action of this drug. A common clinical feature of sunitinib-related acute cholecystitis is acalculous cholecystitis [3-5], which was also observed in our two cases, whereas gallbladder stones are found in 90% of patients with acute cholecystitis [7]. Previous trials have reported the side effects of sunitinib treatment to be reversible and tolerable. The percentage of patients with grade 3 or 4 side effects did not exceed 10% [8, 9]. In the present cases, neither of the patients exhibited right upper quadrant pain with a positive Murphy’s sign. Therefore, it is possible that the detection of acute cholecystitis was delayed, thus resulting in the need to perform cholecystectomy in order to control acute cholecystitis.

As sunitinib is widely used in the treatment of RCC and GISTs, the oncology community should be alerted to be aware of this uncommon and life-threatening adverse event.

### Conclusion

We herein described two cases of sunitinib-related acute acalculous cholecystitis in patients with clear cell RCC who ultimately underwent cholecystectomy to control their symptoms. Attention must be paid to the potential for sunitinib-related acute cholecystitis, which, although uncommon, can be life-threatening.
